# Overlapping transcriptional expression response of wheat zinc-induced facilitator-like transporters emphasize important role during Fe and Zn stress

**DOI:** 10.1186/s12867-019-0139-6

**Published:** 2019-09-23

**Authors:** Shivani Sharma, Gazaldeep Kaur, Anil Kumar, Varsha Meena, Jaspreet Kaur, Ajay Kumar Pandey

**Affiliations:** 1 National Agri-Food Biotechnology Institute (Department of Biotechnology), Sector 81, Knowledge City, Mohali, Punjab 140306 India; 2 University Institute of Engineering and Technology, Panjab University, Sector 25, Chandigarh, Punjab 160015 India

**Keywords:** Micronutrient uptake, *Triticum aestivum* L., Zinc transport, Biofortification, Iron deficiency

## Abstract

**Background:**

Hexaploid wheat is an important cereal crop that has been targeted to enhance grain micronutrient content including zinc (Zn) and iron (Fe). In this direction, modulating the expression of plant transporters involved in Fe and Zn homeostasis has proven to be one of the promising approaches. The present work was undertaken to identify wheat zinc-induced facilitator-like (ZIFL) family of transporters. The wheat *ZIFL* genes were characterized for their transcriptional expression response during micronutrient fluctuations and exposure to multiple heavy metals.

**Results:**

The genome-wide analyses resulted in identification of fifteen putative *TaZIFL*-like genes, which were distributed only on Chromosome 3, 4 and 5. Wheat ZIFL proteins subjected to the phylogenetic analysis showed the uniform distribution along with rice, *Arabidopsis* and maize. In-silico analysis of the promoters of the wheat *ZIFL* genes demonstrated the presence of multiple metal binding sites including those which are involved in Fe and heavy metal homeostasis. Quantitative real-time PCR analysis of wheat *ZIFL* genes suggested the differential regulation of the transcripts in both roots and shoots under Zn surplus and also during Fe deficiency. Specifically, in roots, *TaZIFL2.3, TaZIFL4.1, TaZIFL4.2, TaZIFL5, TaZIFL6.1* and *TaZIFL6.2* were significantly up-regulated by both Zn and Fe. This suggested that ZIFL could possibly be regulated by both the nutrient stress in a tissue specific manner. When exposed to heavy metals, *TaZIFL4.2* and *TaZIFL7.1* show significant up-regulation, whereas *TaZIFL5* and *TaZIFL6.2* remained almost unaffected.

**Conclusion:**

This is the first report for detailed analysis of wheat *ZIFL* genes. ZIFL genes also encode for transporter of mugineic acid (TOM) proteins, that are involved in the release of phytosiderophores to enhance Fe/Zn uptake. The detailed expression analysis suggests the varying expression patterns during development of wheat seedlings and also against abiotic/biotic stresses. Overall, this study will lay foundation to prioritize functional assessment of the candidate ZIFL as a putative TOM protein in wheat.

## Background

Cereal crops are important targets for enhancing micronutrient content, including iron (Fe) and zinc (Zn) in the developing grains. From human nutritional point of view, these micronutrients play an important role in growth and development, cognitive and immune impairment, and in gene regulation [1–3]. Therefore, researches worldwide are identifying diverse approaches to generate micronutrient rich food crops. Apart from a nutritional point of view, Fe and Zn are also essential minerals for plant development and various biochemical functions [4, 5]. Multiple metal specific transporters and regulators show co-expression and could share common signaling features including the response towards Zn, Fe or other metals. Several specific transporters that are involved in the uptake and translocation of Fe and Zn, inside the plant have been described previously [6–11]. Efficient micronutrient uptake by plants is a concerted effort of major genes belonging to the different families of transporters that include, but are not limited to zinc–regulated transporter, iron–regulated transporter family, the natural resistance associated macrophage protein family, yellow-stripe 1-like (YSL) subfamily of the oligopeptide transporter superfamily, Ca^2+^-sensitive cross complementer 1 (CCC1) family [12–16].

Many evidences are gathering that suggest an important role of major facilitator superfamily (MFS) clan of transporters, yet the identification of specific candidate genes has been always a bottleneck. Earlier, MFS-zinc-induced facilitator-1 like (ZIFL) genes, were identified and its role was assessed in different stresses including Zn homeostasis [17]. Role of ZIFL was largely unaddressed in plants until the detailed inventory from model plant *Arabidopsis thaliana* and in crops like *Oryza sativa* [17, 18]. Since, the first identification of the three *ZIFL* in *Arabidopsis* referred to as *AtZIF1* (AT5G13740), *AtZIFL1* (AT5G13750) and *AtZIFL2* (AT3G43790) evidences are accumulating for their role specifically in Zn homeostasis [17]. Subsequently, multiple *ZIFL* genes from monocot such as rice were identified and the presence of high numbers was correlated with the genome duplication events. It was also speculated that plant ZIFL proteins might perform redundant function that is imperative by their overlapping gene expression response during changed regime of Fe and Zn [19]. In addition to that, the rice ZIFL genes were also functionally characterized to be a transporter of mugineic acid (TOM), a phytosiderophore involved in strategy-II mode of Fe uptake via roots [20, 21]. They are involved in efflux of phytosiderophores including deoxymugineic acid (DMA), mugineic acid (MA) or nicotianamine (NA) have been characterized in rice and are reported to be tissue-specific including shoot–root junction or in roots [19, 21]. In rice seeds a positive co-relation was observed in NA and DMA levels with the Fe and Zn content especially in embryo tissue [22]. These functions of TOM family genes suggest their importance in the maintenance of micronutrient homeostasis through secretion of DMA/MA and thereby help to translocate them into the sink areas of plants and is characteristic of Strategy-II mode of Fe uptake [21]. Furthermore, the plant *ZIFL* genes were also reported for their involvement in potassium homeostasis and their ability to transport transition metal such as cesium [23]. These studies provide a valuable clue for ZIFL function that is not just limited to transport of important micronutrients.

Hexaploid wheat (*Triticum aestivum* L.) is an important crop for the developing countries where, the suboptimal levels of grain Zn and Fe have been reported. Initiatives to enhance multiple micronutrients including Zn and Fe are being undertaken by either gain of function approach or RNAi mediated gene silencing [24–26]. Earlier, using wheat grain transcriptome data it was confirmed that a higher proportion of transcripts were present in abundant amount that are known to be involved in transport activity (GO:0005215) [27]. Therefore, such studies provided the framework to investigate new resources and genes that could be of immense value to address the uptake and remobilization of micronutrients in cereal grains like wheat. To provide impetus in this direction, inventory of wheat MFS-ZIFL needs to be explored to identify important candidate genes.

In the current work, 35 ZIFL homoeologs representing fifteen genes from hexaploid wheat were identified that showed their restricted distribution only at three chromosomes viz. 3, 4 and 5. Phylogenetic analysis revealed a uniform distribution of wheat ZIFL sequences in multiple clades along with rice, *Arabidopsis* and *Zea mays*. Detailed characterization of the *ZIFL* genes for their motif composition, promoter sequences and their expression under Fe limiting and Zn surplus condition and other heavy metals was also performed. Our data indicate that the wheat *ZIFL* show overlapping expression response during Fe deficiency and Zn excess condition. Few of the wheat *ZIFL* gene expression remained unaffected by the presence of heavy metals. Overall, characterization of crop ZIFL transporters could result in identifying specific candidate/s that could be used further to modulate specific Fe-Zn uptake in crop plants such as wheat.

## Results

### Genome-wide identification and phylogenetic analysis of wheat ZIFL

**Fig. 1:**
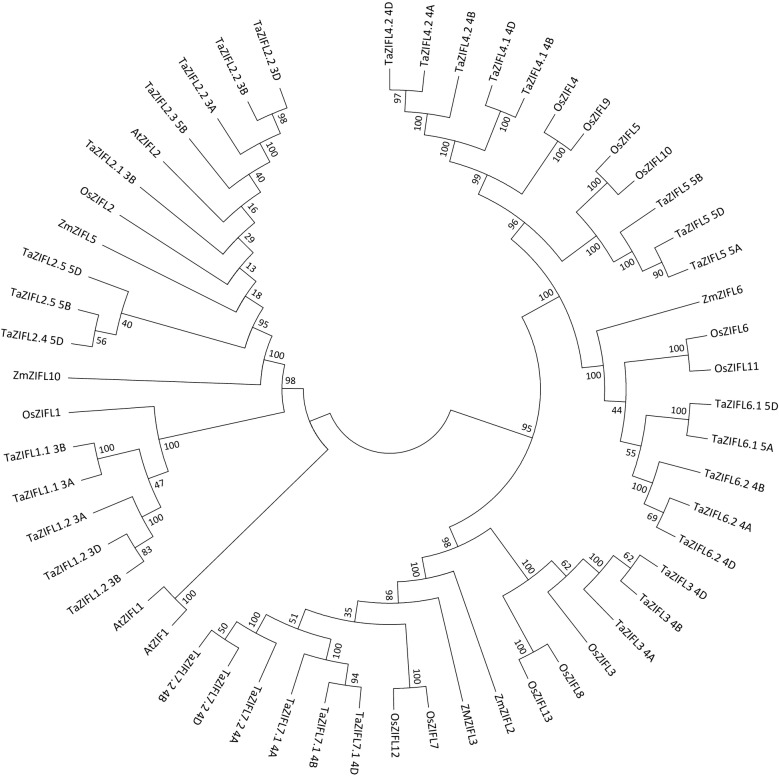
Phylogenetic analysis of wheat ZIFL protein sequences. The analysis was performed by using 35 ZIFL protein sequences from wheat, thirteen from *Oryza sativa*, seven from *Zea mays* and three from *Arabidopsis*. Rooted phylogenetic tree was constructed by using Neighbour-joining method using MEGA7 software with 1000 bootstrap replicates

In order to identify wheat *ZIFL* genes and to gain insight for possible evolutionary relationship, two complementary approaches were used. This includes, first performing genome-wide sequence search of MFS_1 family using Pfam BLAST (PF07690), followed by homology-based analysis with previously reported *ZIFL* genes in different plant species using Ensembl database. These approaches resulted in the identification of one hundred seventy-nine sequences and to further validate their identity sequences were checked and searched for MFS_1 domain through Pfam and conserved domain databases (CDD-NCBI) (Additional file 1: Table S1). These sequences were then used to build phylogenetic tree with previously known ZIFL protein sequences from different plants (Additional file 1: Table S1 and Additional file 2: Figure S1). The arrangement of tree suggested a distinct clade for the ZIFL cluster when compared to the remaining MSF_1 proteins. This indicates that ZIFL is a distinct group of MFS transporters that are tightly clustered (Additional file 2: Figure S1). Further, this distribution was confirmed through signature sequences that are specific to ZIFL proteins. The presence of either of these signature sequences validated the distribution of ZIFL proteins. Two wheat specific signature sequences included (i) W-G-x(3)-D-[RK]-x-G-R-[RK] (found in all except in TaZIFL2.5-5D) and (ii) S-x(8)-[GA]-x(3)-G-P-x(2)-G-G with an exception of A instead of G at 10th position of (ii) signature in TaZIFL2. Furthermore, sequences similar to ZIFL specific cysteine (Cys) and histidine (His) signatures were also used for identifying TaZIFL. (iii) C-[PS]-G-C, absent in 6 TaZIFL sequences (TaZIFL2.5-5D, TaZIFL 3-4B, TaZIFL 4.2-4B, TaZIFL 5-5D, TaZIFL 7.1-4B, TaZIFL 7.2-4B) probably due to missing sequence information and (iv) [PQ]-E-[TS]-[LI]-H-x-[HKLRD] (an insertion of ETLYCRHEHRYSIFISLD sequence within the motif was found in TaZIFL7.2-4A) [18]. These signatures guided identification of specific wheat ZIFL from the rest of the MFS_1 member. Such analysis resulted in confirmation of a total of thirty-five wheat ZIFL sequences, including individual *TaZIFL* genes and their respective homoeologs from different wheat sub-genomes (Additional file 3: Table S2). To check the distribution along with other plant species, ZIFL protein sequences from *O. sativa*, *Z. mays* and *Arabidopsis* were used to build a rooted phylogenetic tree through the NJ method (Fig. 1). Because of the genome duplication events in wheat, the genes are likely to show multiple alleles of a single gene. Hence the resulted 35 putative wheat *ZIFL* represent 15 genes after distribution with respective homoeologs (Fig. 1). To provide the uniform nomenclature, *TaZIFL* genes were named according to their respective closest known orthologs from rice. Among the wheat ZIFL proteins TaZIFL4.1 showed highest homology with TaZIFL4.2 of 95.2 percentage identity. When a cross species comparison was done, the maximum identity of 87 percent was shown by TaZIFL2.2-3D and AtZIFL2. With rice, the highest percentage identity of 87 was observed for wheat ZIFL2.2-3A and OsZIFL2. Divergence was observed among TaZIFL3-4B and OsZIFL13 with percentage identity of 50.

### Molecular structure and genome organization

**Fig. 2:**
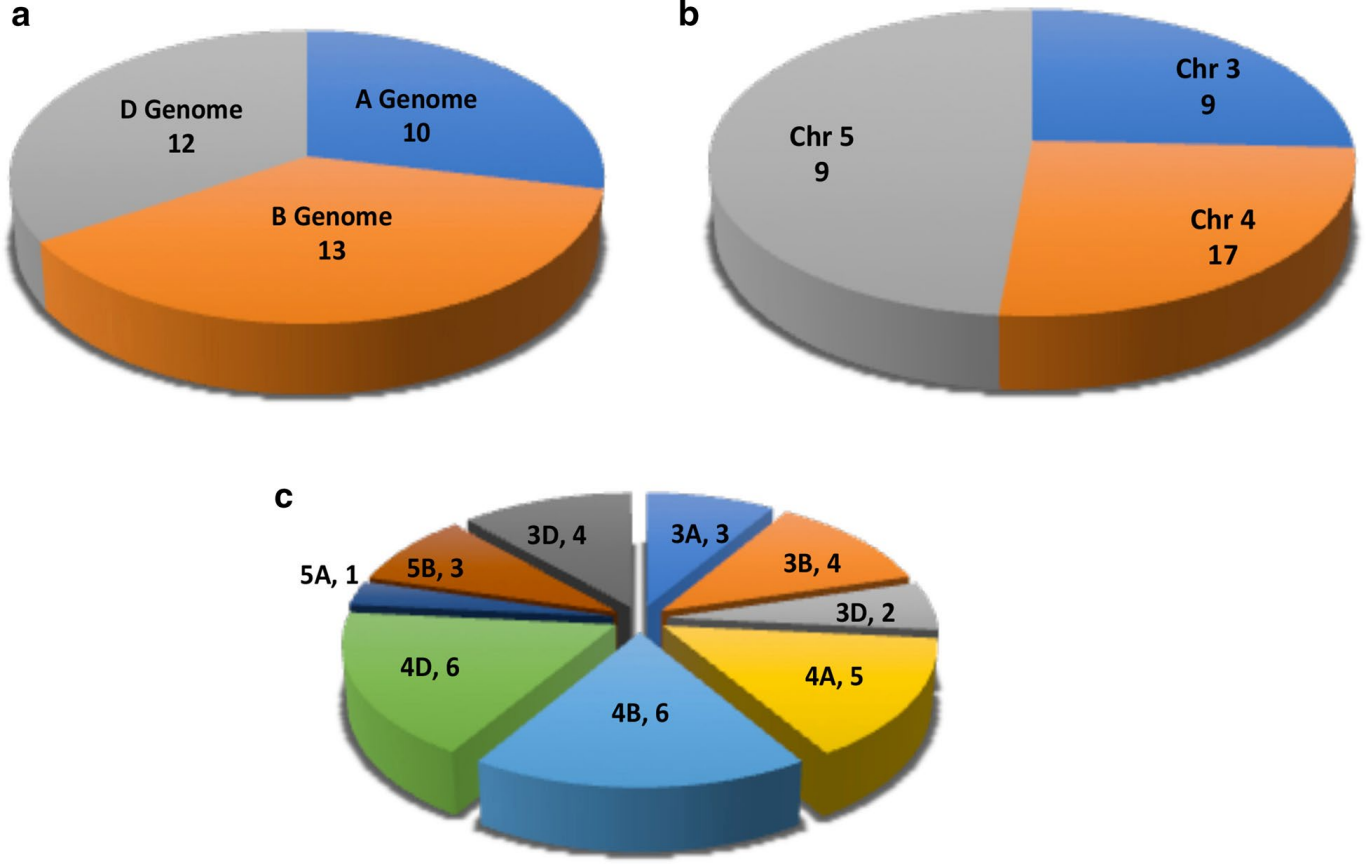
Genomic and chromosomal distribution of wheat ZIFL genes on wheat genome. Distribution of 35 TaZIFL across: **a** A, B and D sub genomes. **b** Wheat chromosomal distribution. **c** Chromosomal distribution shared on different Chromosomes and sub-genomes

**Fig. 3:**
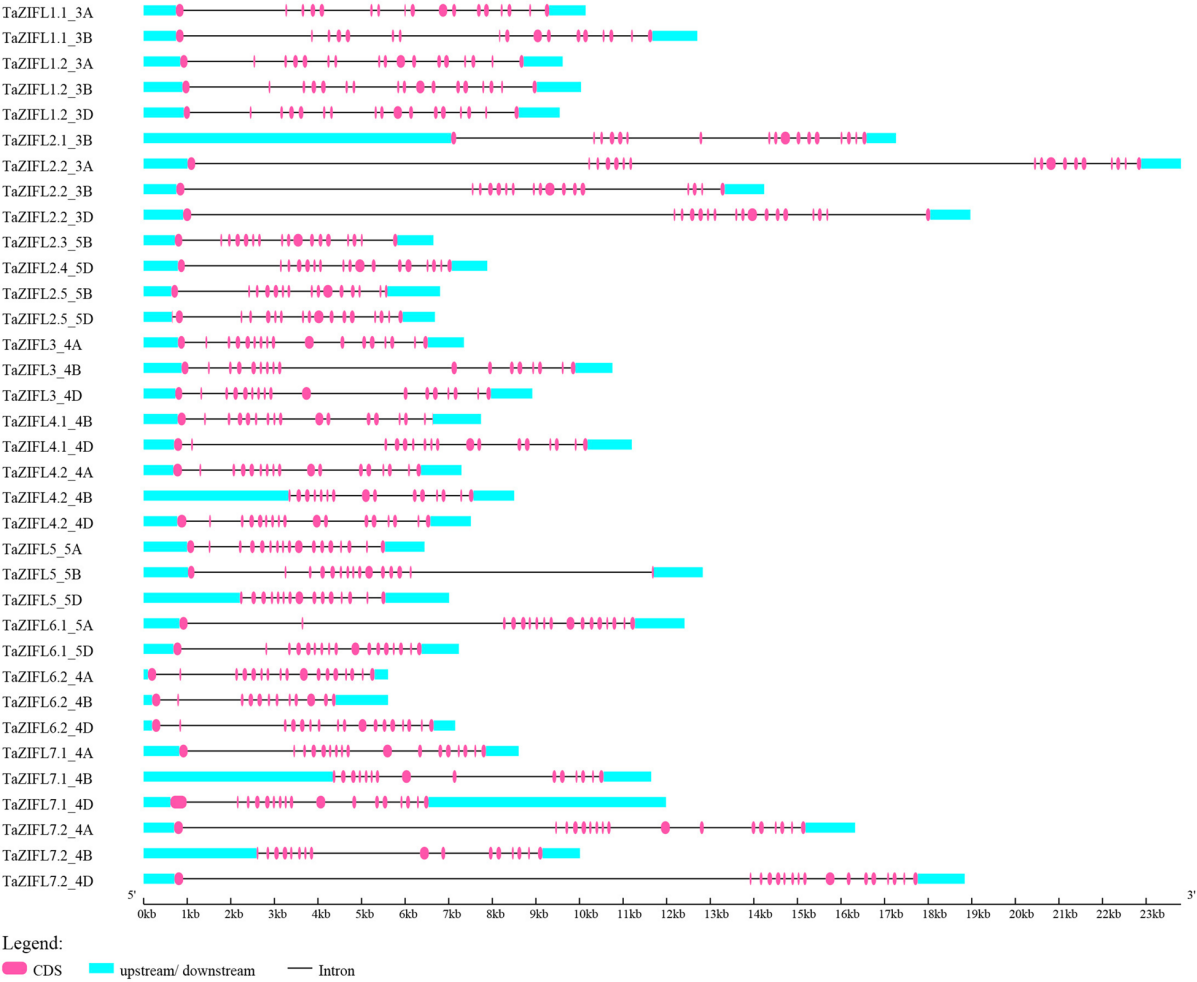
Intron exon arrangement and protein conservation. The intron-exon structure was obtained using Gene Structure Display Server (GSDS 2.0:) Pink boxes and black lines depict the introns and exons, respectively

The predicted protein length of the identified wheat ZIFL sequence ranged from 300 to 562 amino acids (Additional file 3: Table S2). In general, most of the wheat ZIFL showed 10–12 predicted trans-membrane (TM) domains as reported in rice [18]. Specifically, 16 wheat ZIFL proteins were predicted to have 12 TM domains, 15 proteins have 10–11 predicted TM domains, 3 proteins were found to have 8–9 TM domains and only one wheat ZIFL has 4 TM domains (Additional file 3: Table S2). Further, the genomic organization analysis revealed the presence of genes in all three A, B and D sub-genomes. Maximum number of genes were found to be present on B and D sub-genome with 13 and 12 genes respectively (Fig. 2a). TaZIFL1.2, TaZIFL2.2, TaZIFL3, TaZIFL5, TaZIFL6.2, TaZIFL7.1, TaZIFL7.2 are present in all three genomes, while TaZIFL2.3 and TaZIFL2.4 are present on only one genome 5B and 5D respectively (Additional file 3: Table S2). The chromosomal distribution mapping revealed *TaZIFLs* to be present only on chromosome 3, 4 and 5 with maximum of 17 sequences on chromosome 4 (Fig. 2b, c). Next, the genomic structure was analyzed and regions corresponding to intron–exons were marked (Fig. 3). *TaZIFL* clustered into the same group and shared almost similar distribution pattern for the number of exon/intron. The intron–exon number varies from 14 to 18 in the respective *TaZIFL* genomic sequences (Fig. 3, Additional file 3: Table S2).

### Protein motif analysis reveals the presence of diverse domains

**Fig. 4:**
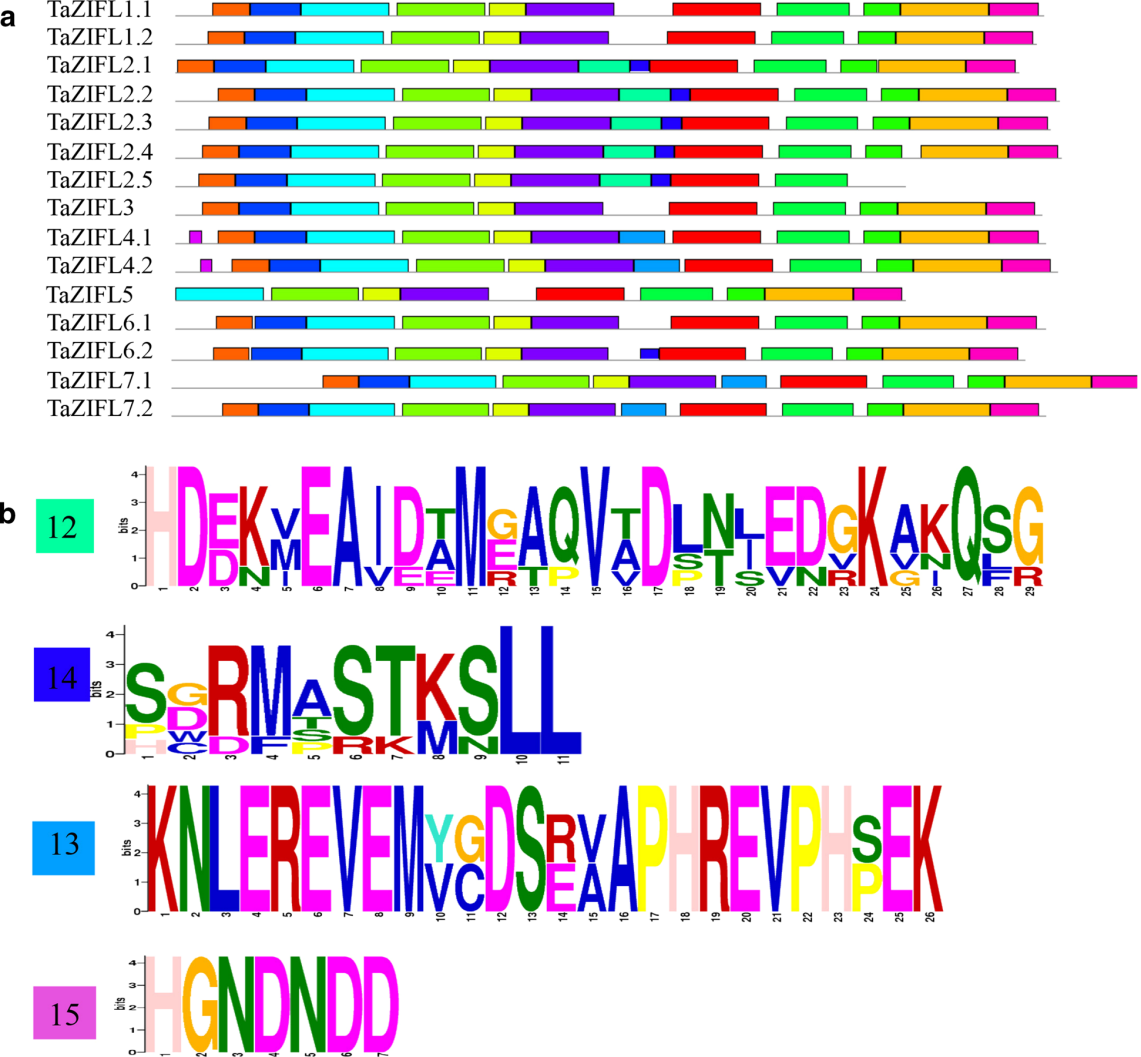
Protein sequence analysis for conserved and unique motifs in wheat ZIFL. **a** Conserved motifs across 15 TaZIFL proteins, as obtained from MEME. Different colors represent distinct motifs. **b** Unique motifs found through MEME for group 2, group 4, as well as TaZIFL6.2

To have an understanding about the similarity, variation in motif composition and distribution of TaZIFL, 15 sequences representing each ZIFL transcript was subjected to MEME analysis. Our analysis revealed the presence of fifteen motifs (Fig. 4a, Additional file 4: Table S3). Out of fifteen motifs, six were conserved throughout all ZIFL, while some lacked few motifs. Four unique and exclusive motifs (12, 13, 14, 15) were identified, which are specific to the respective group. Motif 14 and motif 12 (Fig. 4b, Additional file 4: Table S3) are specific to TaZIFL2.1, TaZIFL2.2, TaZIFL2.3, TaZIFL2.4 and TaZIFL2.5, which indicated that TaZIFL2 members might share similar functions. Motif 14 was also present in TaZIFL6.2. Another set of unique motifs, mentioned as motif 13 and 15 was found in TaZIFL4.1 and TaZIFL4.2, which may indicate probable different function from rest of TaZIFLs. The canonical MFS signature WG[V/M/I][F/V/A/I]AD[K/R][Y/I//H/L]GRKP was majorly present in the cytoplasmic loop between TM2 and TM3 (Additional file 2: Figure S2, Additional file 5: Table S4) as well S-x(8)-G-x(3)-G-P-[A/T/G]-[L/I]-G-G as anti-porter signature mainly in TM5. The results suggest that ZIFL proteins share unique signatures and high similarity indicating they are a distinct group of MFS family. Presence of conserved signatures Cysteine (Cys)-containing motif CPGC reported previously were also present in most of the wheat ZIFL proteins [18]. The absence of these motifs was observed in TaZIFL2.5_5D, TaZIFL 3_4B, TaZIFL 4.2_4B, TaZIFL5_5D, TaZIFL7.1_4B and TaZIFL7.2_4B. This might be because of missing sequence information. This motif was found to be present in the cytoplasmic N-terminal loop for TaZIFL groups 2, 4, 5, 6 and in the non-cytoplasmic N-terminal loop for groups 1, 3 and 7 (Additional file 2: Figure S2 and Additional file 5: Table S4). Another conserved histidine (His)-containing motif PET[L/I]H showed its presence in the cytoplasmic loop between TM domains ranging from 2 and 3 to 6 and 7, with highest between 6 and 7 TM domains (Additional file 2: Figure S2).

### Analysis of conserved *cis*-elements in the promoter of wheat *ZIFL* genes

To find the molecular clues that could regulate the expression of wheat *ZIFL* transcripts, the 1.5 kB promoter region of the all identified wheat *ZIFL* genes was explored. Our analysis revealed a large number of cis-elements in the promoter of wheat ZIFL. Predominantly, the promoters were enriched with the presence of the core binding site for iron-deficiency responsive element binding factor 1 (IDEF), iron related transcription factor 2 (IRO2) and heavy metal responsive element (HMRE) (Additional file 6: Table S5). The presence of these promoter elements suggests that wheat *ZIFL* genes might respond towards the presence of heavy metals and to important micronutrients like Fe and Zn. Interestingly, IDE1 cis-element was present on promoters of all the respective wheat ZIFL genes suggesting that they could respond to Fe deficiency conditions. Few of these promoters consist of multiple such cis-elements suggesting their diverse function in plants (Additional file 6: Table S5).

### Expression patterns of wheat *ZIFL* genes under Zn and Fe stress

**Fig. 5:**
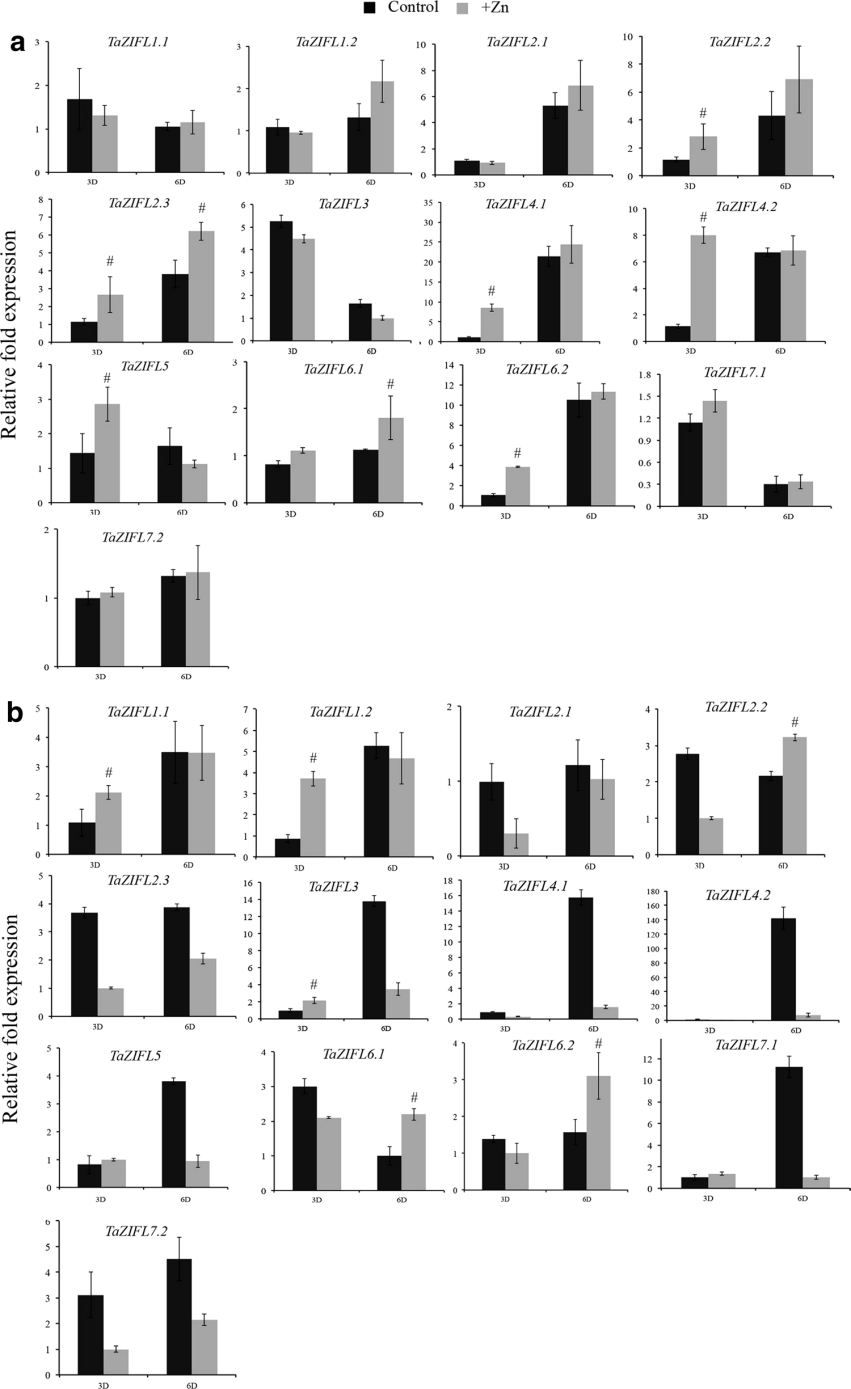
Relative gene expression levels of wheat ZIFL transcripts under +Zn condition. Gene expression profiles of wheat *ZIFL* genes were studied in **a** roots and **b** shoots. Total RNA was extracted from the wheat seedlings subjected to three and 6 days of treatments +Zn. The roots and shoots samples were collected, and qRT-PCR was performed on the DNA free RNAs. A total of 2 µg of RNA was used for cDNA synthesis. C_t_ values were normalized against wheat *ARF1* as an internal control. Data represents mean of three biological replicates each treatment containing 12–15 seedlings. Vertical bars represent the standard deviation. # on the bar indicates that the mean is significantly different at p < 0.05 with respect to their respective control samples

ZIFL are primarily known to respond towards Zn excess (+Zn), therefore experiments were performed to study the gene expression of wheat *ZIFL* in roots and shoots. The qRT-PCR analysis suggested tissue specific expression response by wheat *ZIFL* genes. A total of eight genes, including *TaZIFL1.2*, *TaZIFL2.2, TaZIFL2.3, TaZIFL4.1, TaZIFL4.2, TaZIFL5, TaZIFL6.1* and *TaZIFL6.2* showed significantly higher expression during one of the time points under Zn surplus condition (Fig. 5). Among them, the fold expression level for *TaZIFL4.1* was highest (~ sevenfold) at 3 days after treatment (DAT) with respect to control roots (Fig. 5a). Few genes like *TaZIFL 1.1, TaZIFL7.1 and TaZIFL7.2* remained unaffected by the +Zn condition in roots. In shoots, *TaZIFL1.1, TaZIFL1.2, TaZIFL6.1* and *TaZIFL6.2* showed significant transcript accumulation either at 3DAT or 6DAT after treatment (Fig. 5b). Notably, *TaZIFL1.2, TaZIFL*6.1 and *TaZIFL6.2* show enhanced transcript accumulation in both the tissues. In contrast, during our experiment the expression of a few wheat *ZIFL* genes showed down-regulated in shoots but not in roots. Our expression data under Zn surplus condition suggested the differential response by wheat *ZIFL* towards the treatment.

**Fig. 6:**
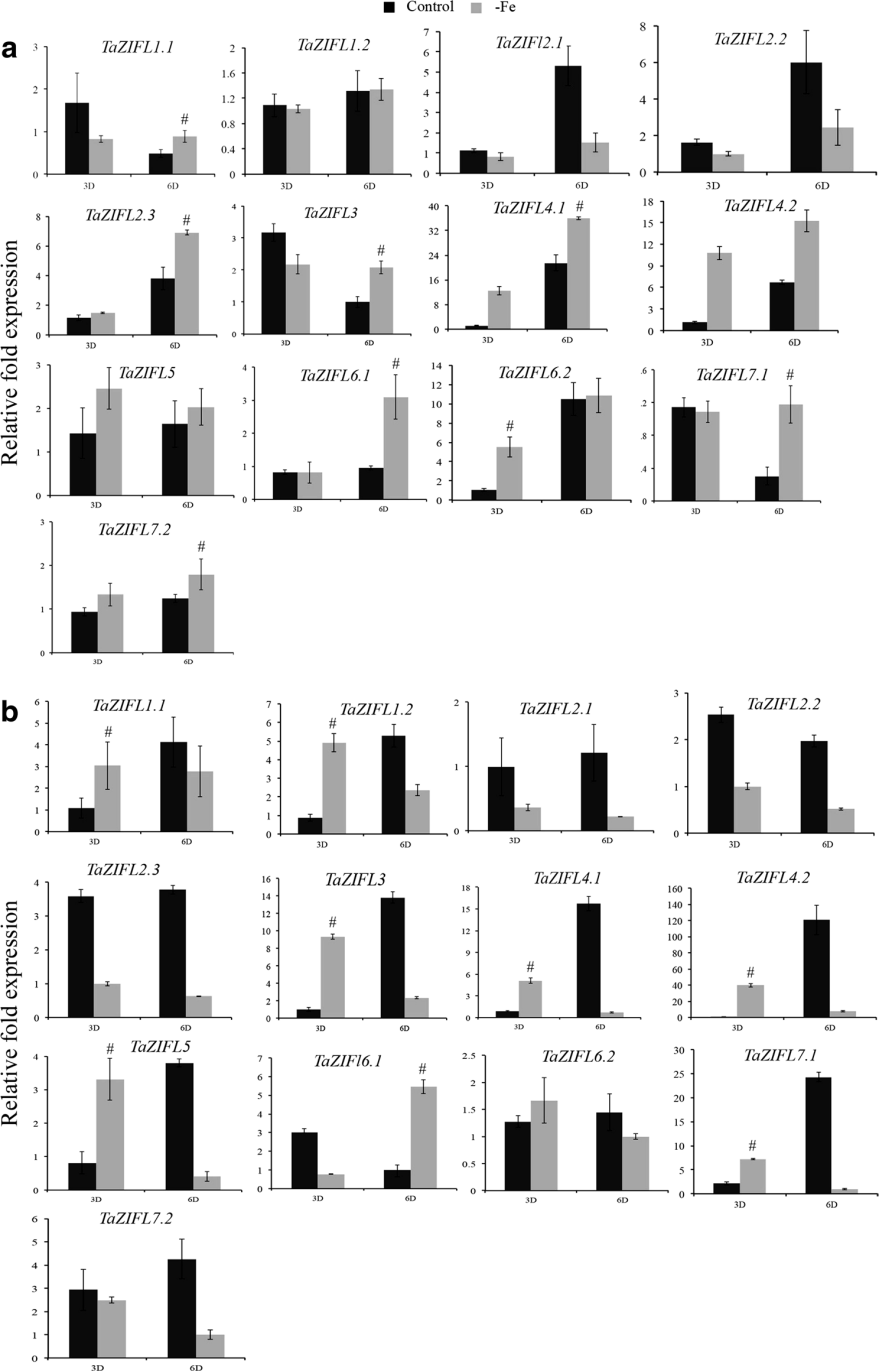
Relative gene expression levels of wheat *ZIFL* genes under −Fe condition. Gene expression profiles of wheat *ZIFL* genes were studied in **a** roots and **b** shoots. Total RNA was extracted from the wheat seedlings subjected to 3 and 6 days of treatments. The roots and shoots samples were collected, and qRT-PCR was performed on the DNA free RNAs. A 2 µg of total RNA was used for cDNA synthesis. C_t_ values were normalized against wheat *ARF1* as an internal control. Data represents mean of three biological replicates with each treatment containing 12–15 seedlings. Vertical bars represent the standard deviation. # on the bar indicates that the mean is significantly different at p < 0.05 with respect to their respective control treatments

Previous evidences indicated that plant *ZIFL* genes not only respond to Zn excess, but are also affected by the Fe limiting conditions [19]. Therefore, expression analysis of wheat *ZIFL* genes was checked in roots and shoots of seedling under Fe starvation (−Fe). Interestingly, in the root expression of *TaZIFL4.1, TaZIFL4.2* and *TaZIFL7.2* show up-regulation during Fe limiting condition at both at 3 and 6 DAT. Out of the remaining genes, *TaZIFL2.3, TaZIFL6.2* and *TaZIFL7.1* show significant transcript abundance at one-time point or the other (Fig. 6a). Interestingly, in shoots *TaZIFL1.1*, *TaZIFL1.2, TaZIFL3, TaZIFL4.1, TaZIFL4.2, TaZIFL5* and *TaZIFL7.1* show up-regulation only at 3 DAT, suggesting their coordinated response in shoots (Fig. 6b). Under −Fe condition, wheat *ZIFL* genes, namely, *TaZIFL4.1* and *TaZIFL4.2* show high transcript accumulation in both roots and shoots. Remaining genes remain unaffected by the Fe stress (Fig. 6b). Overall, our expression data suggested that indeed wheat ZIFL respond to the Fe limiting condition, thereby suggesting a common interlink of this gene family during Zn and Fe homeostasis.

### Candidate *TOM* genes show expression response to the presence of heavy metals

Phylogenetic arrangement of the wheat MFS-ZIFL proteins along with the rice also revealed the possible wheat homologs for TOM transporters. Thus, based on the clade distribution and the percentage identity with the rice TOM (TOM1-OsZIFL4, TOM2-OsZIFL5 and TOM3-OsZIFL7), wheat TOM transporters were identified as TaZIFL4.1/TaZIFL4.2, TaZIFL5 and TaZIFL7.1/TaZIFL7.2. Rice TOM1 and TOM2 falls in the same sub-clade along with wheat along with TaZIFL5 and TaZIFL6, therefore we included them to study their response in presence of heavy metals.

**Fig. 7:**
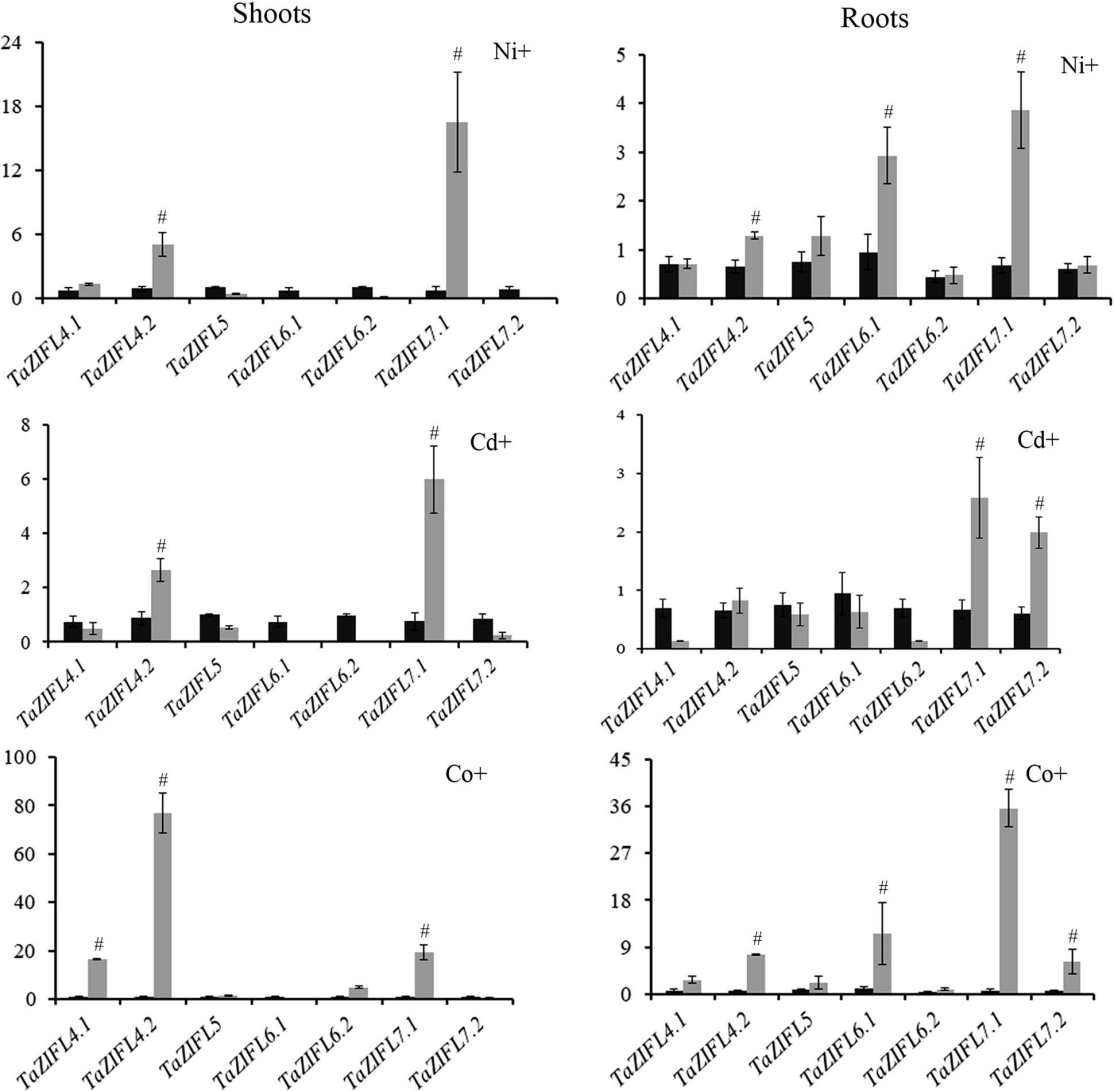
qRT-PCR expression analysis of shoots and roots of wheat seedlings exposed to multiple heavy metals (Ni, Co and Cd). Five days old wheat seedlings were exposed to the mentioned heavy metals for the period of 14 days. Total RNA was extracted from the treated and control samples and 2 µg of RNA was used to construct the cDNA. C_t_ values were normalized against wheat *ARF1* as an internal control. Fold expression values were calculated relative to the control tissue of the mentioned wheat ZIFL. Vertical bars represent the standard deviation. # represent the significantly difference at p < 0.05 with respect to their respective control treatments

Our promoter analysis of wheat *ZIFL* genes indicates the presence of multiple HMRE suggesting that few of these genes could respond to the heavy metals (Additional file 6: Table S5). Due to the importance of *TOM* genes in micronutrient mobilization the expression of these transcripts in wheat seedlings (shoots and roots) was studied after exposure to heavy metals such as Co, Ni and Cd. During our experiment all the seedlings showed phenotypic defects when exposed to heavy metals (data not shown). Our expression analysis suggested that wheat ZIFL genes show metal specific responses. For example, *TaZIFL4.2* and *TaZIFL7.1* showed significant up-regulation in both roots and shoots when exposed to any of the metals tested (Fig. 7). In contrast, the transcripts of *TaZIFL5* and *TaZIFL6.2* remained unaffected under these heavy metals. Expression of *TaZIFL7.2* showed almost no change in the presence of Ni in either of the tissues, yet it was specifically up-regulated in roots when exposed to Cd or Co. Similarly, *TaZIFL6.1* showed significant upregulation only in roots upon exposure to Ni and Co (Fig. 7). Overall, these data indicate the influence of specific heavy metals on the expression of wheat ZIFL genes in a tissue dependent manner.

### Expression of wheat ZIFL transcripts in different wheat tissues and during stress condition

**Fig. 8:**
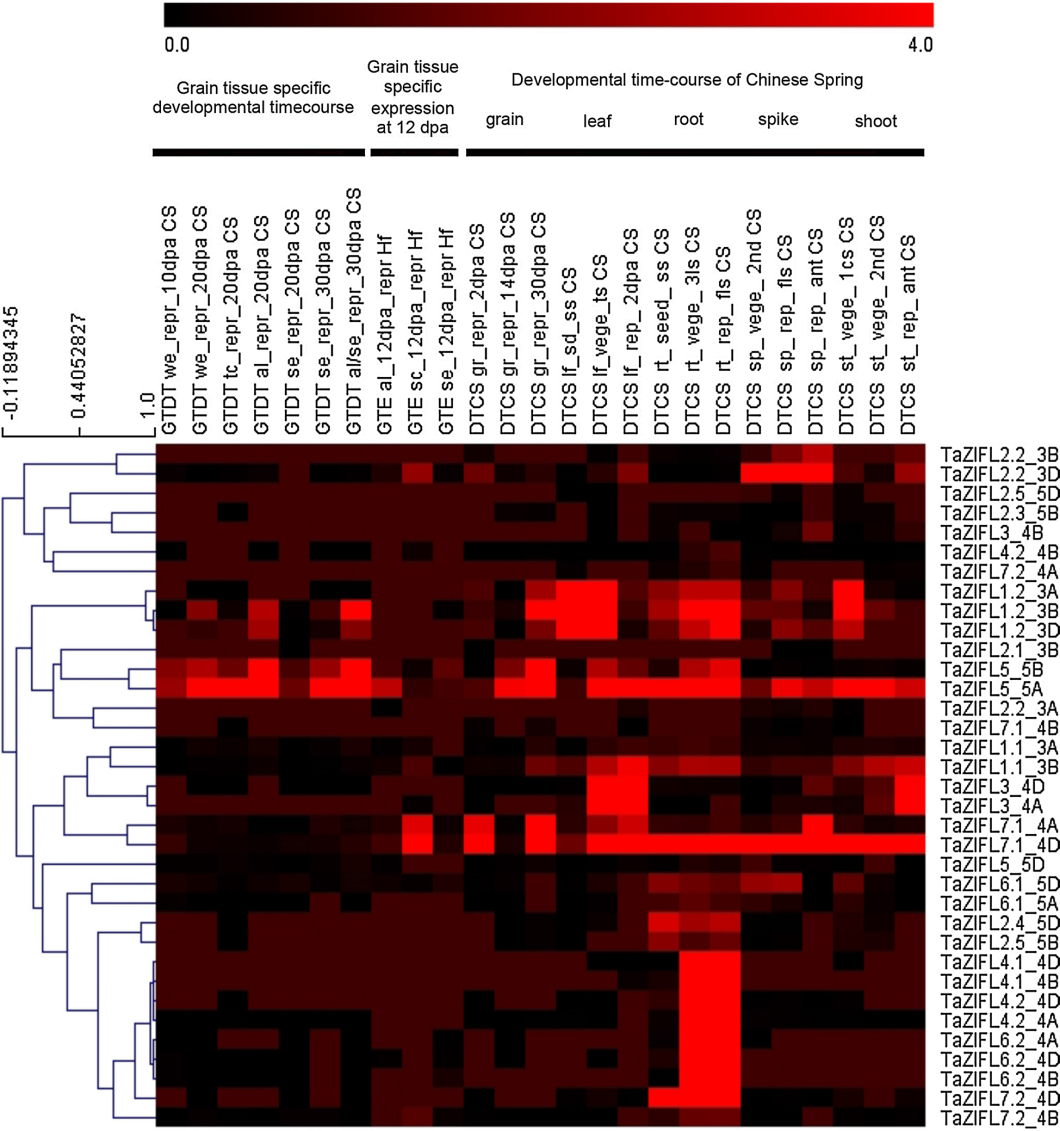
Heat map for relative expression of putative *TaZIFL* genes in different tissues and at multiple developmental stages. Heatmaps were generated using expression values from expVIP database for grain, leaf, root, spike and shoot tissues. Green to red color change depicts increase in transcript expression, as shown by the color bar

Analysis of *ZIFL* genes was also performed in different wheat tissues and developmental stages by using qRT-PCR and transcript expression data. Developing grains are an important reservoir for micronutrients, therefore expression studies were carried out for putative wheat TOM genes during the grain maturation. Transcriptional expression of these genes was checked during grain development (7, 14, 21 and 28 days after anthesis-DAA). Our qRT-PCR analysis of putative TOM genes during the grain development showed differential expression pattern with majority of candidate genes (Additional file 2: Figure S3). In general, most of the putative candidate TOM genes showed high expression at the late phase of the grain maturation i.e. 21 and 28 DAA. In addition to this, wheat expression browser and expVIP (http://www.wheat-expression.com/) was used to extracted the expression values as Transcript per millions (TPM). *TaZIFL* expression values in different tissues (aleurone-al, starchy endosperm-se, seed coat-sc, leaf, root, spike, shoot) and various developmental stages were extracted (Additional file 7: Table S6) and depicted as a heatmap (Fig. 8). In reference to grain tissue developmental time course (GTDT) [27], highest expression was seen for *TaZIFL1.2* (3B, 3D) and *TaZIFL5* (5A, 5B), with an increase in expression in “al” at 20 dpa and “al and se” at 30 dpa. In the expression values during grain tissue specific expression (at 12 dpa) [28], *TaZIFL1.2* was not expressed, but like GTDT study, *TaZIFL5* (5A, 5B) was expressed in “al” as well as “se” Fig. 8). While for sc tissue, *TaZIFL2.2*-*3D* and *TaZIFL7.1* (4A, 4D) had the highest expression when compared to other *ZIFL* genes. For the tissue specific expression response *TaZIFL2.2* was abundant in spike, *TaZIFL1.2* in leaf and root. TaZIFL5 was predominantly expressed in all the tested tissue, including leaf, shoot, spike and shoot. The transcripts exclusively expressed in root were TaZIFL2.4-5D, TaZIFL2.5-5B, TaZIFL6.1-5A, and TaZIFL7.2-4D, with high induction of TaZIFL4.1 (4B, 4D), TaZIFL4.2-4A, TaZIFL6.2 (4A, 4B, 4D) for three-leaf and flag leaf stage as compared to the seedling stage. Highest expression induction was seen for TaZIFL4.2-4D. In addition, the highest expression overall in five tissues was observed for TaZIFL1.2 (3A, 3B, 3D) in leaves for seedling as well as tillering stage, TaZIFL2.2-3D in spike, TaZIFL3-4A in leaf, TaZIFL5-5A in grain, TaZIFL7.1-4D in grain and leaf.

For abiotic stress, while no major changes were observed for TaZIFLs, TaZIFL4.1 (4B, 4D) and TaZIFL4.2 (4A, 4D) were found to be significantly downregulated by ~ 14-fold under phosphate deficiency, while TaZIFL6.2-4D was downregulated by threefold (Additional file 2: Figure S4a, Additional file 7: Table S6). Under heat, drought and heat-drought combined stress (Additional file 2: Figure S4a, Additional file 7: Table S6), TaZIFL7.1 (4A, 4D) and TaZIFL7.2-4D were induced by up to ~ sevenfold and ~ twofold respectively, whereas TaZIFL1.2 (3A, 3B, 3D) and TaZIFL5 (5A, 5B) were downregulated by 6 and 7.5-fold, respectively. No significant changes in the *TaZIFL* gene expression were observed for *Fusarium* head blight infected spikelets (Additional file 2: Figure S4b, Additional file 7: Table S6). For *Septoria tritici* infected seedlings, while a ~ twofold induction was observed for *TaZIFL1.2* (3A, 3B) after 4 days of induction, prolonged infection (13 days), resulted in its downregulation. Other ZIFLs showing changed expression were *TaZIFL1.2*-*3D* and *TaZIFL2.2*-*3A* (> twofold up-regulation), *TaZIFL3*-*4B* (up to 2.7-fold downregulation). *TaZIFL1.2* (3A, 3B, 3D) were also downregulated (up to ~ fourfold) in seedlings with stripe rust infection, while only *TaZIFL1.2*-*3D* was downregulated under powdery mildew infection. These expression data suggest that specific ZIFLs are differentially regulated under infection conditions and show perturbed expression under abiotic stresses.

## Discussion

### Wheat ZIFL proteins as putative phytosiderophore efflux transporters

The current work was undertaken to build the inventory of wheat ZIFL. Since wheat is a hexaploid species with three genomes therefore, we expect a high number of transcripts encoding for a particular gene family. Our analysis resulted in the identification of 35 *ZIFL* gene sequences corresponding to fifteen individual genes from hexaploid wheat. A unique observation made for the wheat ZIFL is that all the genes are restricted to chromosome 3, 4 or 5 only (Fig. 2). This study and the previous preliminary report, led to the identification of a total of fifteen *ZIFL* genes with *TaZIFL1.2*, *TaZIFL2.2*, *TaZIFL3*, *TaZIFL4.2*, *TaZIFL5*, *TaZIFl6.2*, *TaZIFL7.1* and *TaZIFL7.2* showing the presence of all the homoeologous (homoalleles) genes [28]. The identified wheat ZIFL proteins belong to the MFS superfamily, thereby containing the canonical MFS signature and antiporter sequence (Additional file 2: Figure S2). Given the high sequence homology, they are named from TaZIFL1 to TaZIFL7 according to their clade distribution in the phylogenetic tree that corresponds to the rice genes. Previously, in rice multiple *TOM* genes were identified as protein belonging to the ZIFL sub-family. Subsequent characterization of these rice *ZIFL* genes led to the identification of functionally active *OsTOM1*, *OsTOM2* and *OsTOM3* [20, 21]. Specifically, TOM2 was shown to be an important internal phytosiderophore efflux transporter whereas, TOM1 was demonstrated to efflux into soil from root cells and thereby involved in metal transport in specific tissue [21]. These observations reinforce the importance of TOM family in translocating metals into the sink areas of the plants [21]. TOM-like genes remain to be identified from hexaploid wheat. Based on the phylogenetic arrangement and the percentage corresponding homologs for the wheat TOM could be *TaZIFL4.1/4.2*, *TaZIFL5* and *TaZIFL7.1/7.2*. Identified wheat ZIFL proteins show localization on the PM, except for TaZIFL4.2-4B, TaZIFL5-5D and TaZIFL7.1-4B. Only TaZIFL4.2-4B, TaZIFL5-5D are putatively localized in vacuolar membrane, thereby making them a potential candidate to assess their tissue specific role in micronutrient mobilization or storage in the cell organelles.

In general, very small number of *ZIFL* genes are being reported from dicot species like *Arabidopsis* and *Vitis vinifera* and *Populus trichocarpa* [18]. Given the complexity of the wheat genome, we anticipated the presence of multiple possible putative phytopsiderophore efflux transporters that need to be functionally characterized in the near future. In rice, the high ZIFL numbers have been accounted due to the lineage-specific expansion by duplication of the gene family [18]. Overall, our analysis identified highest number of *ZIFL* genes reported till date from any monocot species.

### Wheat ZIFL genes display overlapping gene expression

Plants undergoing metal stress result in series of signaling events that largely include reprogramming of transcripts to overcome the toxic effects. In plants, excess of Zn also results in the generation of reactive oxygen and nitrogen species [29]. The abundance of multiple membrane proteins is increased by the presence of either excess Zn or Fe deficiency. In line with the previous studies, our data also confirmed the overlapping expression response of wheat *ZIFL* genes [30]. Additionally, under Fe limiting conditions, induction of Zn responsive genes could be an important step towards limiting the non-specific transport activity of transporters which are primarily induced for Fe deficiency. ZIFL are well known for their response towards the presence of excess Zn [17]. Our study revealed that multiple wheat ZIFL genes responded to excess Zn, either in shoots or roots in a temporal manner. Interestingly, most of the wheat *ZIFL* genes show the presence of Fe responsive cis-element IDE1. IDE1 is one of the primary cis-elements that respond to Fe limiting conditions [31]. Therefore, we studied the expression of wheat *ZIFL* genes under –Fe condition. Multiple ZIFL genes showed specific response towards Fe deficiency, suggesting that the deficiency response shown by ZIFL transcripts could be mediated by transcription factors like IDE1. Expression of the few of the ZIFLs like *TaZIFL1.1*, *TaZIFL1.2*, *TaZIFL4.2* and *TaZIFL6.2* were also affected by both Fe and Zn in either root or shoot. RNA-seq expression analysis of Fe deficiency in wheat roots for 20 days was checked for the expression of wheat ZIFL genes. At the late stage of Fe deficiency TaZIFL4.1 and TaZIFL4.2 show highest expression (Additional file 2: Figure S5) [32]. These results suggest that few of these ZIFL genes might be involved in the overlapping pathways of Fe and Zn homeostasis. Such commonality of Zn and Fe homeostasis were reported earlier and are also evident from our work. This may suggest the overlap of the common network of transcription factors involved in Fe and Zn homeostasis. In our study, the promoters of *TaZIFL1.2*, *TaZIFL2.3, TaZIFL7.1* and *TaZIFL7.2* showed the presence of IRO2 binding domains that has been previously speculated to be the link between Fe and Zn homeostasis [18]. Nonetheless, only *TaZIFL1.2* respond to −Fe and +Zn stress that is restricted only to shoots. Previously, it was also shown that expression of Arabidopsis ZIF1 remained unaffected in the presence of sub-inhibitory levels of Cd or Cu [17]. Based on the expression response of ZIFL genes during in the presence of heavy metals it seems that *TaZIFL5* and *TaZIFL6.2* could be one of the best candidate genes for the further studies, as both the genes remained unaffected. Nevertheless, careful selection of candidate gene must be done to minimize the cotransport of other undesired metals during micronutrient uptake.

In addition to their anticipated role in Fe and Zn homeostasis, their role in root development has been also proven. Plant ZIFL transporters have been reported to regulate stomatal movements by means of polar auxin transport, thereby modulating potassium and proton fluxes in *Arabidopsis* [23]. Analysis of the expVIP data suggested high expression of wheat *ZIFL* genes (*TaZIFL7.1*-*4A* and *4D*) under drought condition. Earlier, *zifl*-*1* and *zifl*-*2* mutants of *Arabidopsis* showed hypersensitivity to drought stress by disruption of guard cells activity [33]. *TaZIFL1.2* is the wheat transporter showing highest expression in leaf and highest homology with *AtZIFL1*, thereby belonging to the same clade in the phylogeny tree. In contrast to the expected function of ZIFL in Fe and Zn homeostasis, a putative role has been demonstrated in plant defense. Maize ZIFL referred as *Zm*-*mfs1* was high induced during plant defense and has been implicated its role for export of antimicrobial compounds during its interaction with the bacterial pathogen [34]. In our study, a strong expression of multiple *ZIFL* genes was observed when infected with multiple pathogens suggesting its important role in providing resistance against fungal pathogens (Additional file 2: Figure S4b).

This study concludes that ZIFL transporters are the important players during the crosstalk of Fe and Zn homeostasis. With the evidence regarding their role as a transporter for NA and MA in crops, ZIFL genes are certainly a priority candidate to address the uptake and remobilization of micronutrients in the cereal grains such as wheat.

## Conclusion

This the first comprehensive study that resulted in the identification of fifteen putative *ZIFL* genes from hexaploid wheat at the homoeolog level. These are the highest number of *ZIFL* genes reported in plant system till date. Wheat ZIFL were characterized for their expression response in seedlings exposed to excess Zn and Fe deficiency. The contrast expression of these ZIFL in presence of heavy metals suggested their functional redundancy and pinpoint importance of a few candidate *ZIFL* genes for their functional validation. Our work identified candidate ZIFL from hexaploid wheat that could be the important target to address new means to enhance micronutrient uptake by targeting phytosiderophore release.

## Methods

### Identification of the MFS_1 family in wheat

To identify the potential members of *ZIFLs* from MFS_1 transporter family in wheat genome, we used two independent approaches. In the first approach, the Pfam number (PF07690) for MFS_1 was used and sequences were extracted from wheat using the Ensembl wheat database. As a complementary approach, known sequences of *ZIFL* genes from *A. thaliana*, *O. sativa* and *Brachypodium distachyon* were retrieved and used for BLAST analysis against the wheat database: Ensembl (http://plants.ensembl.org/Triticum_aestivum/) to retrieve the sequences. The identified *MFS_1* superfamily was validated through the domain search in CDD-NCBI database (https://www.ncbi.nlm.nih.gov/Structure/cdd/wrpsb.cgi) [35].

### Identification, classification, and chromosomal distribution of wheat *ZIFL* genes

To identify putative *TaZIFLs* genes among MFS_1 superfamily sequence, phylogenetic tree was constructed with known ZIFLs from different plants that separated the ZIFL cluster from the rest of the MFS_1 superfamily members. This distribution of genes in ZIFL cluster was validated through the presence of ZIFL specific signature sequences. To construct the phylogenetic tree one hundred seventy-nine wheat MFS_1 superfamily protein sequences were retrieved from Pfam no. (PF07690). In addition to that, thirteen-protein sequence of ZIFL from *O. sativa*, five from *Zea mays* and three from *Arabidopsis* were used. Identified TaZIFLs were named according to their corresponding rice orthologs having maximum number of ZIFL reported. The name indicates corresponding ortholog from rice followed by chromosomal and genomic location e.g. *TaZIFL3*-*4A*, *TaZIFL3*-*4B* and *TaZIFL3*-*4D* represent three homoeologous of *TaZIFL* genes in chromosome four of all the three A, B and D sub-genomes and it is an ortholog of *OsZIFL3.* To know the evolutionary relationship among ZIFL proteins, phylogenetic analysis was performed along with other plant species (*O. sativa*, *Z. mays* and *Arabidopsis)*. All the proteins were aligned through MUSCLE algorithm and a rooted phylogenetic tree was used to construct with the Neighbor-joining (NJ) method using MEGA7 software with 1000 bootstrap replicates [36]. To determine the distribution of *ZIFL* genes in the wheat chromosomes, the position of each *ZIFL* gene was obtained using wheat Ensembl database.

### Analysis of conserved domains, gene arrangements and subcellular localization of wheat ZIFL

The divergence and conservation of motifs in wheat ZIFL proteins were also identified by using MEME (Multiple Expectation Maximization for Motif Elicitation) program version 5.0.2 (http://meme-suite.org) [37] with a maximum motif width, 50; maximum number of motifs, 15; and minimum motifs width, 6. Gene Structure Display Server (GSDS 2.0) [38] was used to analyze the gene structure. Individual wheat ZIFL CDS and corresponding genomic DNA were aligned to identify the intron–exon arrangement. Using Expasy Compute PI/MW online tool (http://us.expasy.org/tools/protparam.html) the predicted isoelectric points and molecular weights of putative TaZIFLs were calculated. To predict terminal ends and number of transmembrane domains, TMHMM (http://www.cbs.dtu.dk/services/TMHMM/) was utilized. The putative protein sequences of ZIFL were further in silico analyzed to predict their subcellular localization by WoLF PSORT (https://wolfpsort.hgc.jp/) prediction program [39].

### Plant material and Fe, Zn and heavy metals treatments

For giving various Zn and Fe treatments, seeds of *Triticum aestivum* cv. C306 (provided by Punjab Agriculture University, Ludhiana, India) were used. Bread wheat cv. C306, has been undertaken for research purpose since it is having a good processing (including chapatti making) quality [40, 41]. Previously this cultivar was also used for Fe deficiency related studies to characterize wheat YSL transporter [16]. For experiments, the seeds were washed with double autoclaved water for the removal of dirt followed by surface sterilization with 1.2% Sodium hypochlorite prepared in 10% ethanol. Seeds were stratified by keeping them overnight in dark at 4 °C on moist Whatman filter papers in a Petri dish. The stratified seeds were further allowed to germinate at room temperature. For each experiment, healthy seedlings were transferred to phytaboxes (12–15 seedlings/phytabox: in three biological replicates) and grown in autoclaved water in growth chamber for 5 days. The endosperm was removed after 5 days and plants were kept in their respective media according to the treatment. Five days old seedlings were subjected to different Zn and Fe conditions and grown in Hoagland media [42] in triplicates supplemented with either 2 µM of Fe(III) EDTA for Fe deficient condition (−Fe) or 200 µM of ZnSO_4_.7H_2_O [18] for the Zn surplus experiment (+Zn). Total S content was adjusted with the macronutrient used in Hoagland media. Seedlings grown in the Hoagland media containing 20 µM of Fe(III) EDTA and 2 µM of ZnSO_4_.7H_2_O were used for control experiments. The roots and shoots of plantlets (5 days post germination) were collected after 3 and 6 DAT along with their respective controls. Every alternate day, the seedlings in the phyta-boxes were supplemented with the fresh media, replacing the old media. For heavy metal treatment the 5 days old plantlets were subjected to cadmium (50 µM CdCl_2_), cobalt (50 µM CoCl_2_), nickel (50 µM NiCl_2_) treatment. Root and shoot samples from each replicate were collected after 15 days of treatment. To study the gene expression during different stages of seed development (7, 14, 21 and 28 DAA), tagging was done for the main spikes of the respective biological replicates at the first DAA. At the indicated time points, developing grains from the tagged spikes were collected and frozen in liquid nitrogen for RNA extraction.

### RNA isolation, cDNA preparation and qRT-PCR analysis

Total RNA was extracted from harvested roots and shoot samples using TRIZOL RNA extraction method. Turbo DNAfree kit (Invitrogen, USA) was used to remove the genomic DNA. RNA samples were then quantified on nanodrop and subsequently, 2 µg of total RNA was used to prepare cDNA by using SuperScript III First-Strand Synthesis System (Invitrogen, USA). For expression analysis qRT-PCR primers were designed from the conserved region of respective *TaZIFL* genes to account for the respective additive gene expression response (Additional file 8: Table S7). Amplicons arising from these primers were also processed for sequencing to avoid any cross amplifications of the *ZIFL* genes. Sequencing of the amplicon revealed gene specific expression except for *TaZIFL2.4* and *TaZIFL2.5*, for which either no amplicons were detected under our condition or were non-specific due to its close homology with other members. Therefore, these two were not considered for the expression studies. 10X diluted cDNA and SYBR Green I (QuantiFast^®^ SYBR^®^ Green PCR Kit, Qiagen, Germany) was used to perform qRT-PCR on 7500 Fast Real-Time PCR System (Applied Biosystems, USA). qRT-PCR was performed using three biological replicates and two to four technical replicates. The relative mRNA abundance was normalized with wheat *ARF1* as mentioned earlier (*ADP*-*Ribosylation Factor,* AB050957.1) [43, 44]. The relative expression was calculated through delta–delta CT-method (2^−ΔΔCT^) [45]. The statistical significance of expression data was determined using student’s t-test (p-value < 0.05).

### In-silico expression analysis

For in-silico expression analysis, a total of 35 *TaZIFL* genes were selected and wheat expression browser, expVIP [http://www.wheat-expression.com/] was used to extract the expression values in the form of TPMs. These values were then used to build heatmaps using MeV software [http://mev.tm4.org/]. While absolute values were used for development and tissue-specific data, fold change values were used for stress conditions where a gene was taken to be upregulated if fold change was greater than 2 and downregulated if less than 0.66. For abiotic stress, expression for two studies, phosphate deficiency [46] and heat, drought and heat-drought stress [47] was studied. In case of biotic stress, the studies considered were [48–50]. For expression of wheat *ZIFL* genes under Fe deficiency experiments RNAseq data, NCBI Project ID PRJNA529036 was utilized for analysis.

## Supplementary information

**Additional file 1: Table S1.** List of 179 wheat sequence IDs extracted for MFS_1 family, Pfam ID: PF07690 using ensembl wheat database.

**Additional file 2: Figure S1.** Neighbor-Joining (NJ) tree for MFS_1 family of proteins from *Oryza sativa, Brachypodium distachyon, Zea mays* and *Triticum aestivum* constructed using MEGA7.0 software with a bootstrap replicate value of 1000. The phylogenetic tree shows ZIFL proteins clustering into a distinct clade within the MFS family. **Figure S2.** Alignments for signature sequences specific for ZIFL proteins, namely, ZIFL MFS signature motif, the anti-porter signature, Cys-containing, His-containing signatures. **Figure S3.** Gene expression analysis of wheat ZIFL transcript during grain development. qRT-PCR analysis of putative wheat TOM genes: *TaZIFL4.1*, *TaZIFL4.2*, *TaZIFL5*, *TaZIFL7.1* and *TaZIFL7.2.* qRT-PCR was performed on cDNA prepared from 2 µg of total RNA as mentioned in methods. Fold expression levels were calculated relative to 7 days after anthesis (DAA) tissue. **Figure S4.** Heat-map depicting relative differential expression of putative wheat ZIFL genes under various abiotic and biotic stresses: **a.** Heat maps of TaZIFL genes under various abiotic (Phosphate starvation, Drought, Heat and Drought-Heat). **b.** Biotic stresses (*Fusarium* heat blight, *Septoria tritici*, stripe Rust and Powdery mildew). Heat-maps were generated using fold change values obtained after processing the expression values from expVIP database. Green color represents down-regulation, black represents no change and red color represents up-regulation. **Figure S5.** Gene expression analysis of wheat ZIFL transcript in root during Fe deficiency. Heatmap showing expression for putative *TaZIFL* genes in roots of control and 20 days post Fe deficiency. Heatmap was generated using MeV software based on the expression values calculated from NCBI Project ID PRJNA529036 [33]. Black to red color change depicts increasing expression, as shown by the color bar.

**Additional file 3: Table S2.** Detailed information for 35 putative wheat ZIFLs identified. Table includes gene IDs, chromosomal locations, CDS and protein lengths, molecular weight and pI for each of the obtained putative *TaZIFLs*.

**Additional file 4: Table S3.** Conserved motifs identified in putative TaZIFL proteins using MEME. The consensus sequence logo, e-values and the number of sequences in which each motif was found are listed.

**Additional file 5: Table S4.** ZIFL Signature motifs (Cys, His, MFS and MFS antiporter) and their locations in all wheat ZIFL proteins. The motif positions were mapped to the TM-HMM predicted TaZIFL protein structures to obtain the location of motifs.

**Additional file 6: Table S5.** Cis-elements of wheat ZIFL genes along with their positions in the promoter. HMRE—heavy metal responsive element, IRO2—iron related transcription factor 2, MRE: metal responsive element, IDE1: iron-deficiency responsive element binding factor 1.

**Additional file 7: Table S6.** Table listing the details and expression values extracted from expVIP for different developmental stages and tissues, as well as fold change values obtained after processing the TPM values for the stress conditions (Abiotic stress: phosphate deficiency; heat, drought, combined heat-drought stress, & Biotic stress: Fusarium heat blight, *Septoria tritici*, stripe Rust and Powdery mildew).

**Additional file 8: Table S7.** List of the primers used during the current study.

## Data Availability

All data generated or analysed during this study are included in this published article [and its additional information files]. Description of a complete protocol are included within the article.

## Abbreviations

CCC1: Ca^2+^-sensitive cross complementer 1
YSL: yellow-stripe like protein
ZIFL: zinc-induced facilitator-1 like gene
CDD: conserved domain database
MFS: major facilitator superfamily
TM: trans-membrane
HMRE: heavy metal responsive element
IRO2: iron related transcription factor 2
IDEF: iron-deficiency responsive element binding factor 1
qRT-PCR: quantitative real time polymerase chain reaction
TOM: transporter of mugineic acid
TPM: transcript per millions
PM: plasma-membrane
DAT: days after treatment
DAA: days after anthesis
