# Supplementary material for: Overlapping transcriptional expression response of wheat zinc-induced facilitator-like transporters emphasize important role during Fe and Zn stress
Source: BMC Mol Biol. 2019 Sep 23;20:22. doi: 10.1186/s12867-019-0139-6 (PMC6757437; doi:10.1186/s12867-019-0139-6)
Supplement: Supplementary file 2 — Additional file 2: Figure S1. Neighbor-Joining (NJ) tree for MFS_1 family of proteins from Oryza sativa, Brachypodium distachyon, Zea mays and Triticum aestivum constructed using MEGA7.0 software with a bootstrap replicate value of 1000. The phylogenetic tree shows ZIFL proteins clustering into a distinct clade within the MFS family. Figure S2. Alignments for signature sequences specific for ZIFL proteins, namely, ZIFL MFS signature motif, the anti-porter signature, Cys-containing, His-containing signatures. Figure S3. Gene expression analysis of wheat ZIFL transcript during grain development. qRT-PCR analysis of putative wheat TOM genes: TaZIFL4.1, TaZIFL4.2, TaZIFL5, TaZIFL7.1 and TaZIFL7.2. qRT-PCR was performed on cDNA prepared from 2 µg of total RNA as mentioned in methods. Fold expression levels were calculated relative to 7 days after anthesis (DAA) tissue. Figure S4. Heat-map depicting relative differential expression of putative wheat ZIFL genes under various abiotic and biotic stresses: a. Heat maps of TaZIFL genes under various abiotic (Phosphate starvation, Drought, Heat and Drought-Heat). b. Biotic stresses (Fusarium heat blight, Septoria tritici, stripe Rust and Powdery mildew). Heat-maps were generated using fold change values obtained after processing the expression values from expVIP database. Green color represents down-regulation, black represents no change and red color represents up-regulation. Figure S5. Gene expression analysis of wheat ZIFL transcript in root during Fe deficiency. Heatmap showing expression for putative TaZIFL genes in roots of control and 20 days post Fe deficiency. Heatmap was generated using MeV software based on the expression values calculated from NCBI Project ID PRJNA529036 [33]. Black to red color change depicts increasing expression, as shown by the color bar. [file 12867_2019_139_MOESM2_ESM.pptx]

## Slide 1
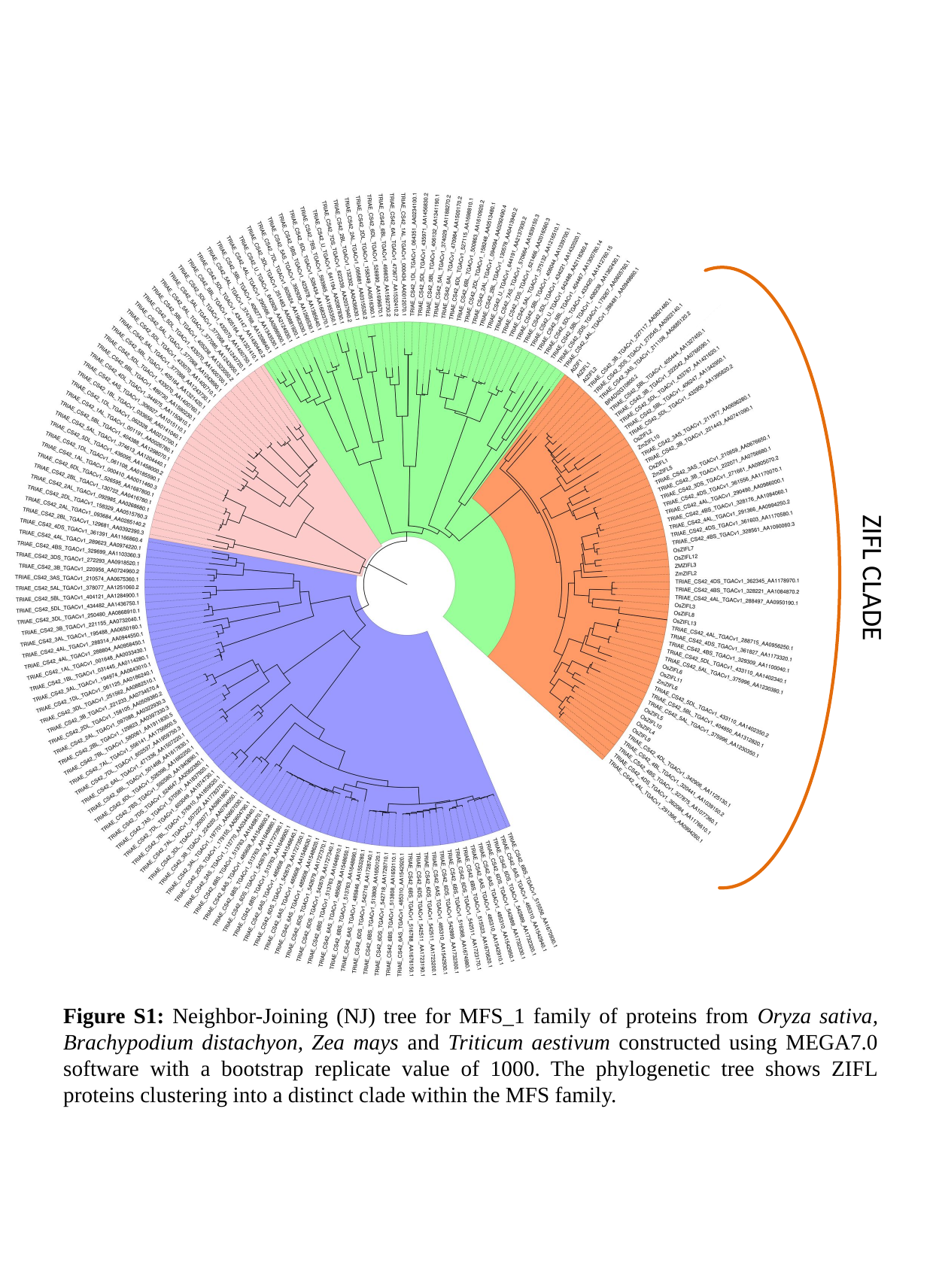

ZIFL CLADE
Figure S1: Neighbor-Joining (NJ) tree for MFS_1 family of proteins from Oryza sativa, Brachypodium distachyon, Zea mays and Triticum aestivum constructed using MEGA7.0 software with a bootstrap replicate value of 1000. The phylogenetic tree shows ZIFL proteins clustering into a distinct clade within the MFS family.

## Slide 2
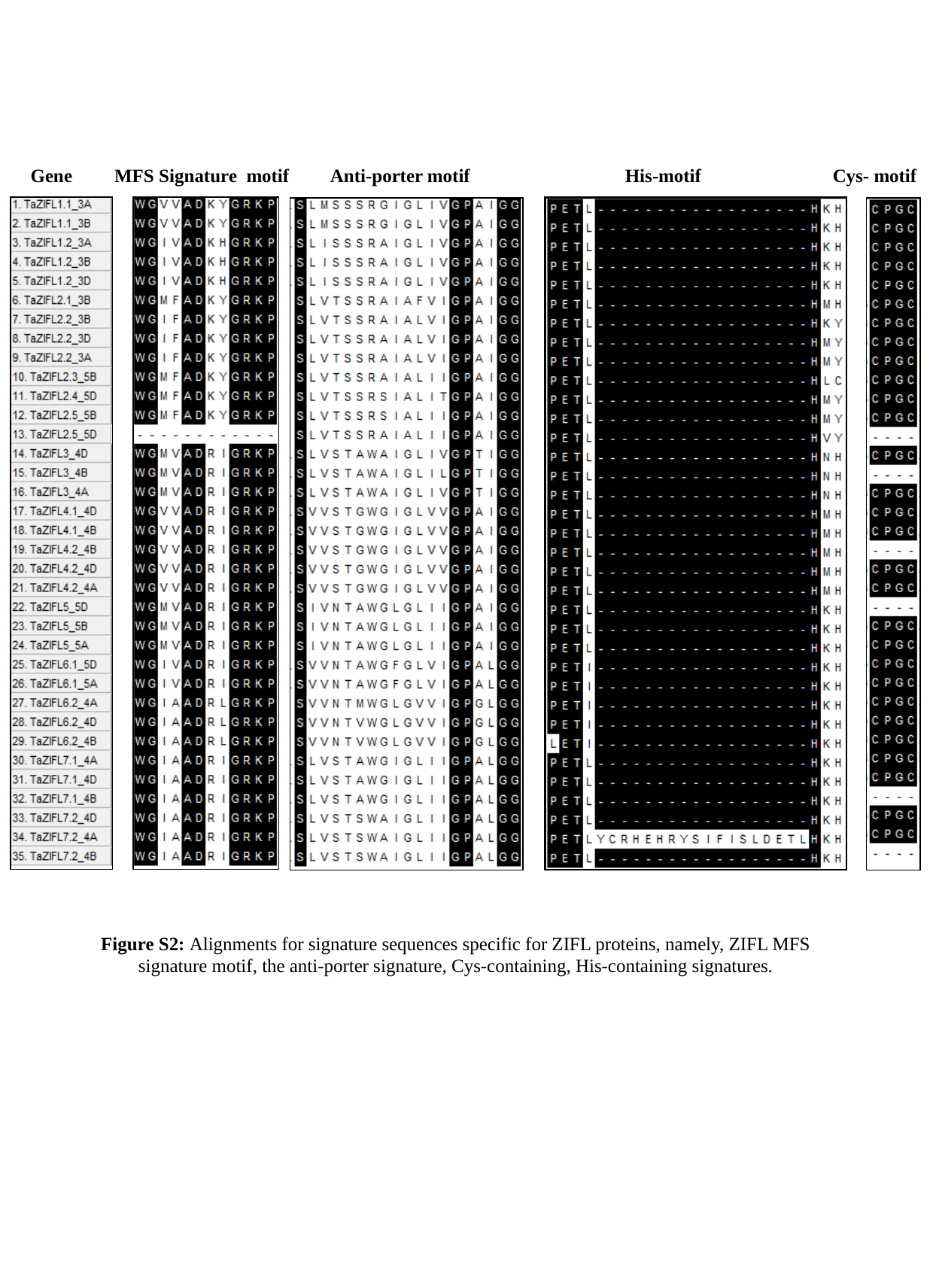

Gene MFS Signature motif Anti-porter motif His-motif Cys- motif
Figure S2: Alignments for signature sequences specific for ZIFL proteins, namely, ZIFL MFS signature motif, the anti-porter signature, Cys-containing, His-containing signatures.

## Slide 3
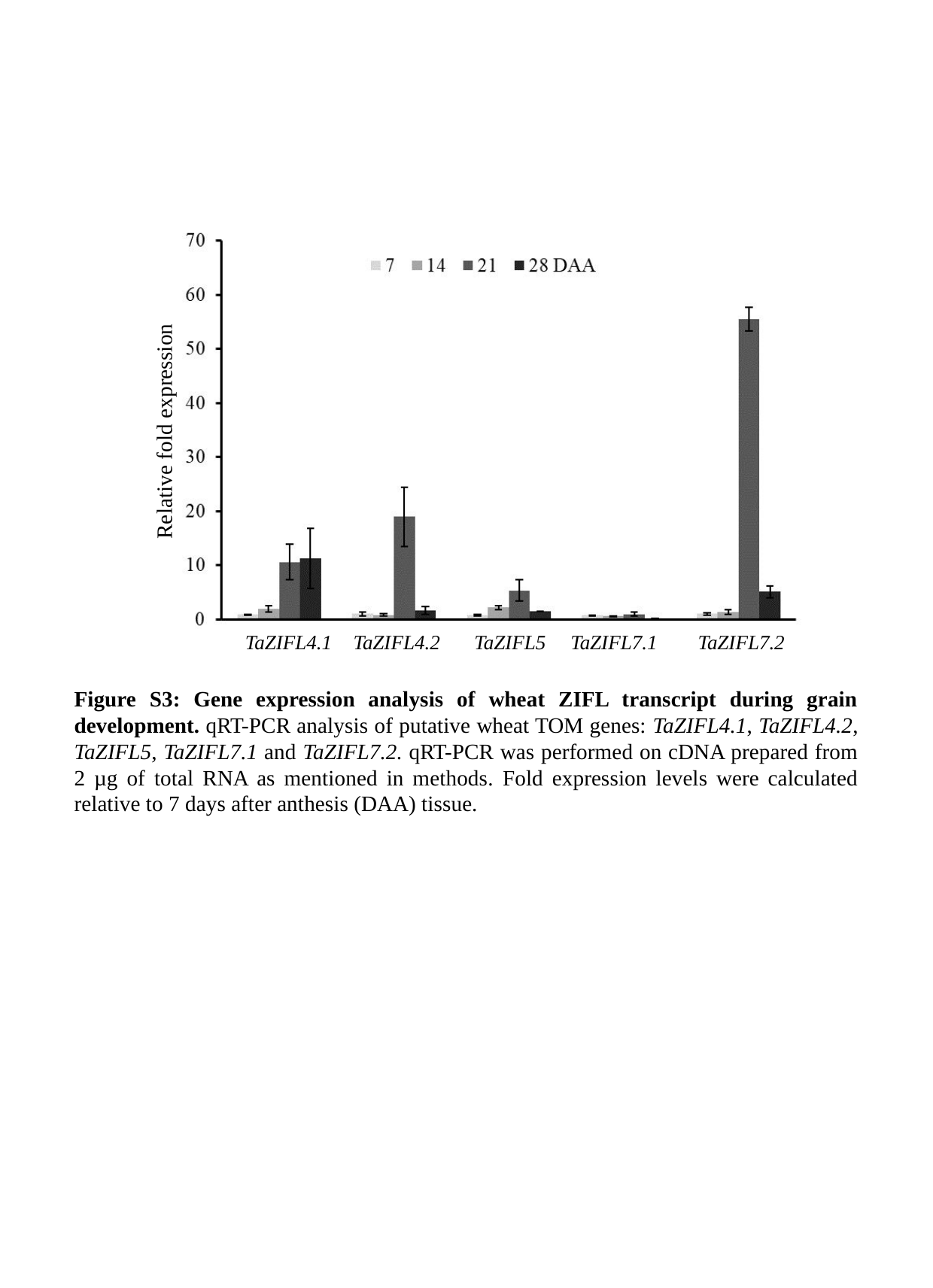

Relative fold expression
TaZIFL4.1
TaZIFL4.2
TaZIFL5
TaZIFL7.1
TaZIFL7.2
Figure S3: Gene expression analysis of wheat ZIFL transcript during grain development. qRT-PCR analysis of putative wheat TOM genes: TaZIFL4.1, TaZIFL4.2, TaZIFL5, TaZIFL7.1 and TaZIFL7.2. qRT-PCR was performed on cDNA prepared from 2 µg of total RNA as mentioned in methods. Fold expression levels were calculated relative to 7 days after anthesis (DAA) tissue.

## Slide 4
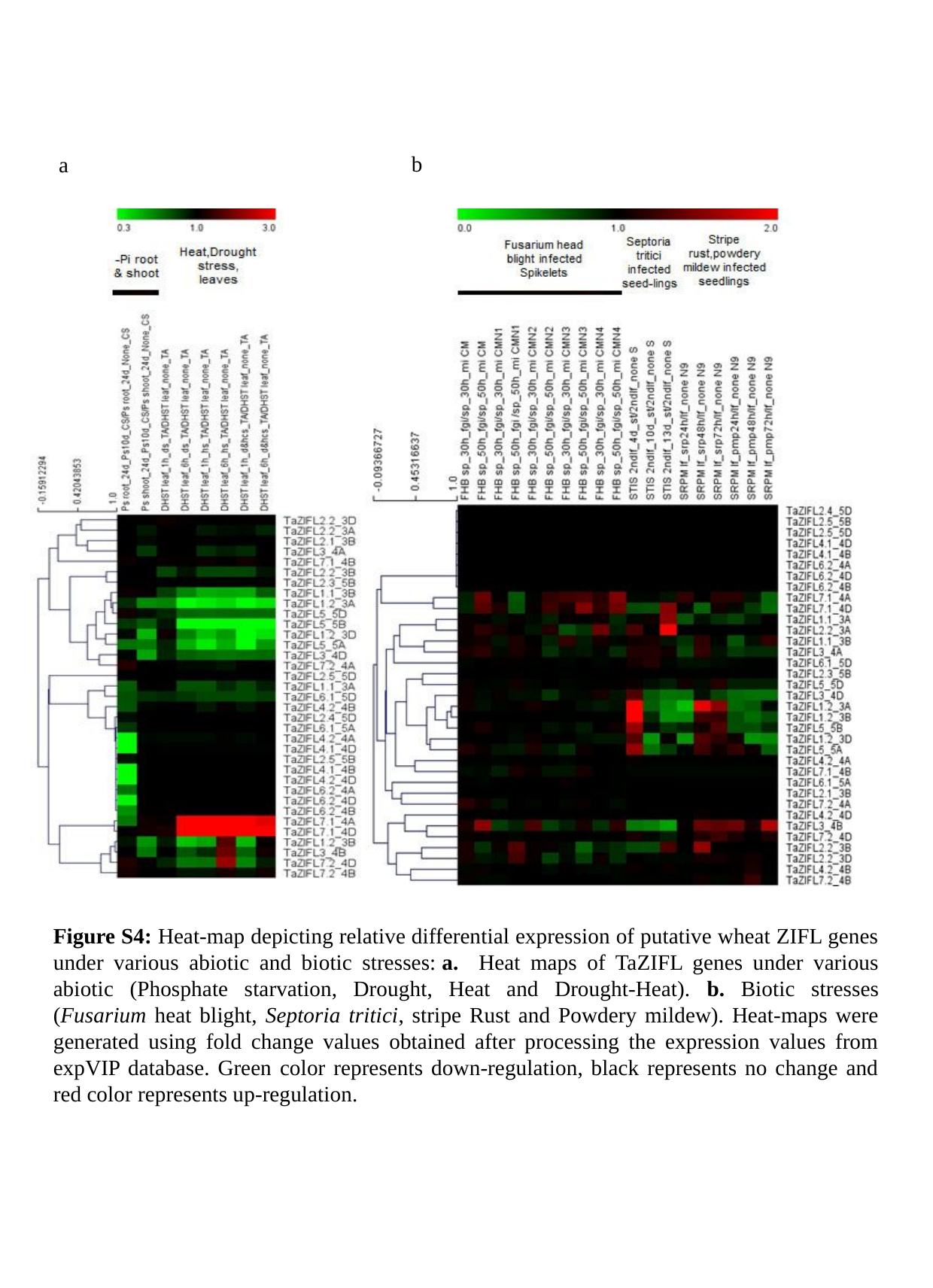

b
a
Figure S4: Heat-map depicting relative differential expression of putative wheat ZIFL genes under various abiotic and biotic stresses: a. Heat maps of TaZIFL genes under various abiotic (Phosphate starvation, Drought, Heat and Drought-Heat). b. Biotic stresses (Fusarium heat blight, Septoria tritici, stripe Rust and Powdery mildew). Heat-maps were generated using fold change values obtained after processing the expression values from expVIP database. Green color represents down-regulation, black represents no change and red color represents up-regulation.

## Slide 5
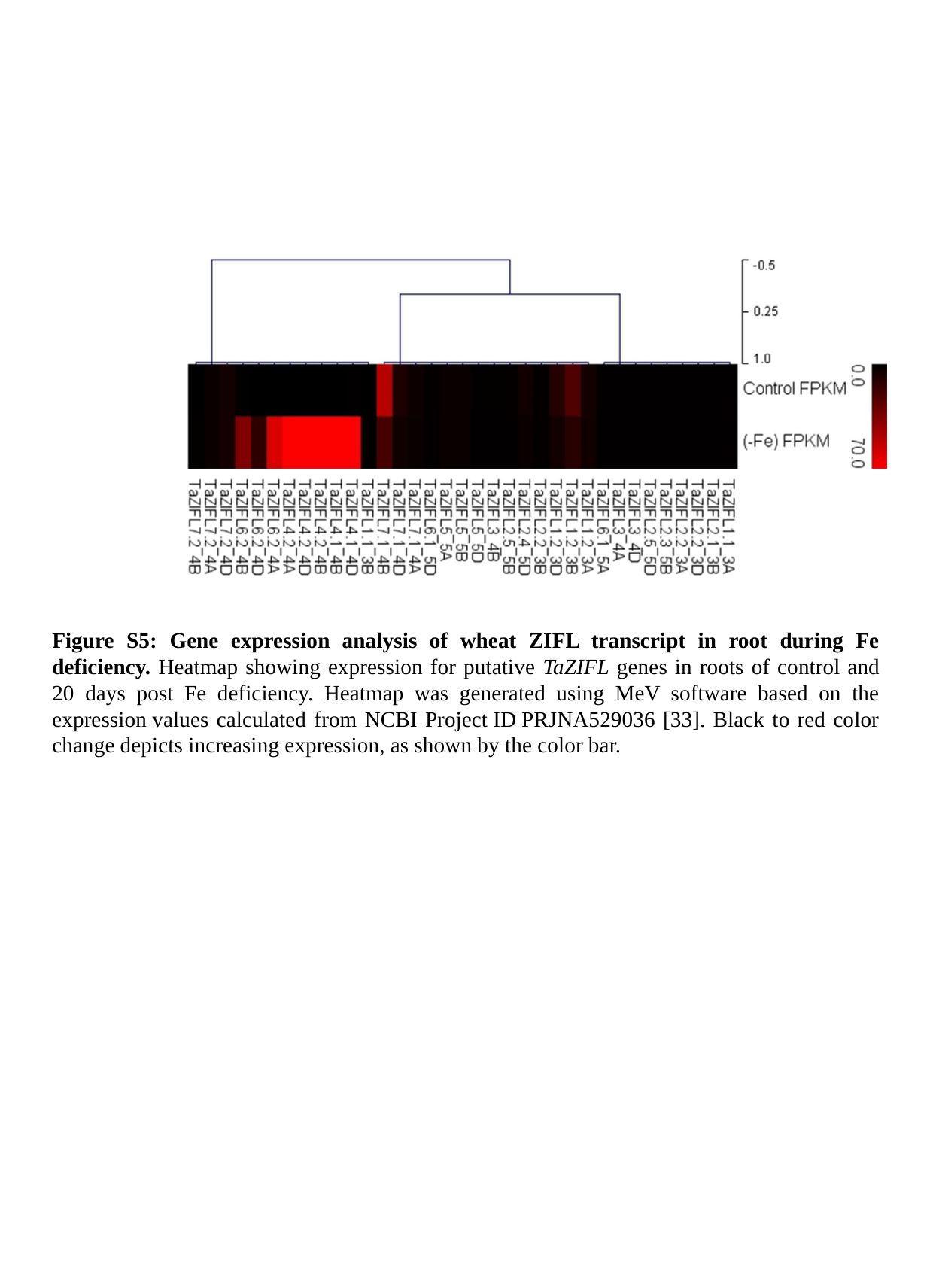

Figure S5: Gene expression analysis of wheat ZIFL transcript in root during Fe deficiency. Heatmap showing expression for putative TaZIFL genes in roots of control and 20 days post Fe deficiency. Heatmap was generated using MeV software based on the expression values calculated from NCBI Project ID PRJNA529036 [33]. Black to red color change depicts increasing expression, as shown by the color bar.
